# Using Competency-Based Digital Open Learning Activities to Facilitate and Promote Health Professions Education (OLAmeD): A Proposal

**DOI:** 10.2196/resprot.4974

**Published:** 2016-07-07

**Authors:** Christos Vaitsis, Natalia Stathakarou, Linda Barman, Nabil Zary, Cormac McGrath

**Affiliations:** ^1^ Karolinska Institutet Department of Learning, Informatics, Management and Ethics Stockholm Sweden; ^2^ Nanyang Technological University Lee Kong Chian School of Medicine Singapore Singapore

**Keywords:** medical education, competency frameworks, technical standards, open learning activities, massive open online courses, learning management systems

## Abstract

**Background:**

Traditional learning in medical education has been transformed with the advent of information technology. We have recently seen global initiatives to produce online activities in an effort to scale up learning opportunities through learning management systems and massive open online courses for both undergraduate and continued professional education. Despite the positive impact of such efforts, factors such as cost, time, resources, and the specificity of educational contexts restrict the design and exchange of online medical educational activities.

**Objective:**

The goal is to address the stated issues within the health professions education context while promoting learning by proposing the Online Learning Activities for Medical Education (OLAmeD) concept which builds on unified competency frameworks and generic technical standards for education.

**Methods:**

We outline how frameworks used to describe a set of competencies for a specific topic in medical education across medical schools in the United States and Europe can be compared to identify commonalities that could result in a unified set of competencies representing both contexts adequately. Further, we examine how technical standards could be used to allow standardization, seamless sharing, and reusability of educational content.

**Results:**

The entire process of developing and sharing OLAmeD is structured and presented in a set of steps using as example Urology as a part of clinical surgery specialization.

**Conclusions:**

Beyond supporting the development, sharing, and repurposing of educational content, we expect OLAmeD to work as a tool that promotes learning and sets a base for a community of medical educational content developers across different educational contexts.

## Introduction

In the past 20 years there has been a growing momentum among higher education institutions to take part in the provision of open educational resources, or teaching, learning, and research resources that reside in the public domain or have been released under an intellectual property license that permits their free use or repurposing by others [1,2]. The recent massive open online courses (MOOC) initiatives have provided insights into how such open material may be scaled up to reach a large number of users. Other initiatives such as the open educational resources programs in the United Kingdom and the IMS global learning consortium [3] have enabled the design and development of teaching and learning resources to be made freely available worldwide ‘’using copyright licenses that promote their use, reuse, and repurposing’’ [1,4]. This process has run concurrently with the emergence of digital learning making substantial advances in medical education [5]. Furthermore, the development and sharing of digital educational and training material has the potential to offer more economically viable options [6]. Most recently and perhaps most notably, the emergence of MOOC platforms has enabled open educational resources that can reach massive numbers of students enabling engagement in learning across professional, social, and geographical borders. This, we believe, also has the potential to foster and scale up medical education by addressing some of the current challenges identified by Mehta et al [7] such as test grades that reflect students’ test performance without verifying the acquired skills and attributes that build the desired competencies [8]. However, one of the challenges to useful open educational resources is the lack of a shared language or sense of purpose between professionals; in addition, material developed in one context may not be readily useful in another [9].

From the instructor's perspective, developing digital learning material usually involves numerous challenges such as cost, time, and resources restrictions [6,10]. Furthermore, the outreach may be limited or local [11], and the variety of learning management systems (LMSs), defined as enterprise-wide and Internet-based systems that integrate a wide range of pedagogical and course administration tools [12]; educational platforms; and technological infrastructures limits reusability, which could reduce the cost of developing the same material at various places in the world and the time spent on lecturing [13]. Technical standards, online content sharing, and content metadata exploitation have partially addressed these challenges [14-17]. It is clear that there is interest in supporting the community in the design process of online educational material for MOOCs and LMSs [18], and the medical education community could benefit from this as well. However, to the best of our knowledge there has been no initiative yet for supporting, exchanging, and displaying this material holistically with a solution that could readily be adopted to address all the stated challenges.

In this proposal we present an idea for how medical education institutions can (1) identify commonalities across competency frameworks, or organized and structured representations of a set of interrelated and purposeful competencies [19]; (2) design open learning activities based on common, identified competencies; and (3) utilize generic technical standards to provide health care professionals with the opportunity to develop and share material in health care professions education.

We propose the development of competency-based digital open learning material, here called OLAmeD (Open Learning Activities for Medical Education). OLAmeD would allow faculty to develop material that could be used in multiple medical education contexts and would be fully functional, either as stand-alone learning modules integrated into a MOOC or as part of an LMS. We expect that OLAmeD, when developed using common competencies and generic technical standards, will promote student learning and support instructors in the process of material production that can be used across borders. With that in place, we believe the incentive to use and reuse material is likely to increase. The use of generic technical standards will give faculty and researchers an opportunity to perform and evaluate interventions on a scale not formerly associated with learning design [6]. Additionally, the development of OLAmeD could promote sharing and professional networking, where the disciplinary community begins to develop OLAmeD in the local context but for global spread, enabling new opportunities for collaboration within disciplines across competency frameworks. The development of OLAmeD will be based on two equally important pillars: identifying commonalities across competency frameworks and using generic technical standards that allow for sharing and reusability.

## Methods

### Identifying Commonalities Across Competency Frameworks

As a first approach, we believe that the comparison of two different competency frameworks, one in the US and one in Europe, which are used to address a common specialization in an undergraduate medical education program, could place us in a position to decide the feasibility of identifying generic outcomes that could be used as the first pillar of OLAmeD.

A first step would compare in a small scale pilot competency frameworks for outcomes used to teach urology as a part of clinical surgery specialization. Our hope is to identify commonalities between competency frameworks that will allow us to identify common outcomes that are applicable across competency frameworks and also to examine the potential to scale up from this initial pilot context to a number of competency frameworks. The urology competency frameworks from two medical schools, one in the United States and one in Europe, have already been mapped and one of the goals we identified to be applicable across both urology frameworks was “Students should have knowledge about the management and treatment of urogenital tumor diseases.” For the comparison between competency frameworks, we will use technical standards developed by the Medbiquitous Consortium [20]. Medbiquitous standards are accredited by the American National Standards Institute and have been implemented by the Association of American Medical Colleges; they are open source and constantly updated to facilitate and advance the health professions education. This provides an integrated approach to expressing, comparing, and verifying the consistency of competencies used through the different standards. We suggest using the Curriculum Inventory standard [21], which is “intended to facilitate the exchange and aggregation of data about health professions curriculum across the continuum of professional education and training” and the Competency Framework standard [22], which “enables users to search for resources addressing a specific competency and determine where competencies are addressed in the curriculum.” We expect to enable comparisons that will eventually promote decisions and actions concerning the potential to identify and target common outcomes.

### Identifying Technical Standards to Allow Reusability and Sharing

To introduce the sharing and reusability of OLAmeD and its integration into different educational platforms, it is essential that OLAmeD includes technically standardized learning activities and content descriptions. For this purpose we see potential in identifying standards that allow the learning content to be organized and distributed with seamless connection of the OLAmeD with different LMS and MOOC platforms, a secure data flow between OLAmeD and other learning environments on predefined standard data elements, and a single sign-on mechanism [14-17]. Further, the identified standards would provide insights into user experiences and record their activities to enable the development and incorporation of metadata that will fully outline OLAmeD.

### Practical Steps Involved in Developing OLAmeD

To summarize the process of developing OLAmeD, we suggest the following steps. First, it would be vital to identify commonalities in competencies from a broad range of curricula (frameworks). As a second step and in order to design a specific learning activity based on the common identified set of competencies it would be necessary to define the learning outcome and choose content, design the digital learning activity that promotes the selected competencies, digitize the learning activity using generic technical standards, and tag the digital learning activity with metadata. In the final step the digital learning activity is ready to be promoted and shared locally and internationally in subject or professional networks.

## Results

Following is a hypothetical case reflecting the expected results and workflow when developing OLAmeD with the above steps. We exemplify using urology as a part of clinical surgery specialization where in the first step we compare urology competency frameworks of curricula in the United States and Europe to identify commonalities. In the second step we design the learning activity based on the common set of competencies where we (1) select the content for the common learning outcome, “Students should have knowledge about the management and treatment of urogenital tumor diseases;” (2) design the digital learning activity using the selected content; (3) digitize the learning activity with a selected technical standard; and (4) tag the digital learning activity with metadata elements that adequately describe it (eg, activity title, activity summary, type of activity, duration, and competency addressed). Finally, we promote and share locally and internationally through established online repositories for OLAmeD exchange.

## Discussion

Today we have the ability to examine medical education curricula and identify overlapping competencies that could be targeted when developing digital learning material. We also have the ability to utilize common technical standards which allow educators to extend the reach of these open learning activities of high quality educational material and make them readily available to multiple users around the globe. These generic technical standards also allow us to monitor student learning and gain insights into the key aspects of learning in medical education. The effort to develop OLAmeD is a worthwhile endeavor if there is a possibility to reach a much broader community of learners and if teachers gain from using learning material produced by others. Current use of existing educational technologies and interoperability standards individually within the context of an institution or organization promotes local development and exploitation but a more collective use is missing. We believe that OLAmeD will add to the current use of educational technology, interoperability standards, and open educational resources because it has the potential to support the efforts of development, sharing, and repurposing of online learning activities and content in medical education and sets a strong base for synergies among collaborating institutions. Additionally, we consider OLAmeD to be an important method for faculty to identify opportunities to develop learning activities for a broader community of learners and potentially engage in a community of faculty education material developers.

## Abbreviations

LMS: learning management system
MOOC: massive open online course
OLAmeD: Open Learning Activities for Medical Education
